# Survival of Mexican Children with Acute Lymphoblastic Leukaemia under Treatment with the Protocol from the Dana-Farber Cancer Institute 00-01

**DOI:** 10.1155/2015/576950

**Published:** 2015-03-26

**Authors:** Elva Jiménez-Hernández, Ethel Zulie Jaimes-Reyes, José Arellano-Galindo, Xochiketzalli García-Jiménez, Héctor Manuel Tiznado-García, María Teresa Dueñas-González, Octavio Martínez Villegas, Berenice Sánchez-Jara, Vilma Carolina Bekker-Méndez, María Guadalupe Ortíz-Torres, Antonio Ortíz-Fernández, Teresa Marín-Palomares, Juan Manuel Mejía-Aranguré

**Affiliations:** ^1^Departamento de Hematología Pediátrica, Unidad Médica de Alta Especialidad (UMAE), Centro Médico Nacional “La Raza”, Instituto Mexicano del Seguro Social (IMSS), Avenida Jacarandas Esquina Vallejo S/N colonia La Raza, 02990 Mexico, DF, Mexico; ^2^Laboratorio de Investigación, Hospital Infantil de México “Federico Gómez”, Secretaría de Salud, Calle Doctor Marquez 162, Colonia Doctores, Delegación Cuauhtémoc, 06720 Mexico, DF, Mexico; ^3^Facultad de Medicina, Universidad Nacional Autónoma de México, Avenida Universidad 3000, 04510 Mexico City, DF, Mexico; ^4^Unidad de Investigación en Epidemiología Clínica, UMAE Hospital de Pediatría, Centro Médico Nacional “Siglo XXI”, IMSS, Avenida Cuauhtemoc 330, 4to Piso Edificio de la Academia Nacional de Medicina, 06720 Mexico, DF, Mexico

## Abstract

Our aim in this paper is to describe the results of treatment of acute lymphoblastic leukaemia (ALL) in Mexican children treated from 2006 to 2010 under the protocol from the Dana-Farber Cancer Institute (DFCI) 00-01. The children were younger than 16 years of age and had a diagnosis of ALL de *novo*. The patients were classified as standard risk if they were 1–9.9 years old and had a leucocyte count <50 × 10^9^/L, precursor B cell immunophenotype, no mediastinal mass, CSF free of blasts, and a good response to prednisone. The rest of the patients were defined as high risk. Of a total of 302 children, 51.7% were at high risk. The global survival rate was 63.9%, and the event-free survival rate was 52.3% after an average follow-up of 3.9 years. The percentages of patients who died were 7% on induction and 14.2% in complete remission; death was associated mainly with infection (21.5%). The relapse rate was 26.2%. The main factor associated with the occurrence of an event was a leucocyte count >100 × 10^9^/L. The poor outcomes were associated with toxic death during induction, complete remission, and relapse. These factors remain the main obstacles to the success of this treatment in our population.

## 1. Introduction

Acute lymphoblastic leukaemia (ALL) is the most common cancer in children and adolescents and is the most frequent cancer in Hispanic children including Mexican children [1]. In developed regions, including North America, Eastern Europe, Australia, New Zealand, and Japan, the survival after 5 years is >90% and the cure rate is 85% [2–6]. These results are now possible because of the implementation of clinical assays involving cooperation between countries [7–11]. However, in developing countries, the survival rate is low, possibly because of the lower quality of medical attention [12]. Several factors are included in the classification of risk: clinical, cytogenetic, immunological, and molecular [13–17]. Despite the availability of these factors for classifying risk, in several developing countries the 1993 National Cancer Institute (NCI) criteria [18] continues to be used for the classification of relapse risk. These criteria take into account the age, leucocyte count, immunophenotype, and the recent response to prednisone, the latter of which has been proven as a strong predictor of response in several groups [19–21].

The Dana-Farber Cancer Institute (DFCI) ALL Consortium is a collaborative group that developed clinical assays from 1985 to 2000. The basis of the treatment is 20–30 dosages of asparaginase during intensification and frequent pulses of vincristine and steroid during maintenance. The survival rates obtained from treatments using this protocol were 82% in the 1980s and 88% in the 1990s [22, 23].

In this paper, we report on our results obtained in a group of Mexican children with acute lymphoblastic leukaemia treated with the DFCI 00-01 protocol modified for our local conditions.

## 2. Patients and Methods

### 2.1. Patients

From August 2006 to December 2010, paediatric patients with ALL who were younger than 16 years of age and who were treated at the paediatric haematology service at the Centro Médico Nacional “La Raza” IMSS were selected. Patients with a diagnosis of mature cells ALL-B mature were not included. The protocol was approved by the institutional committees, and informed consent was obtained from the parents of each patient.

### 2.2. Risk Groups

The patients were stratified according to their risk as standard risk (SR) or high risk (HR). SR was defined as patients 1–9.9 years old, leucocyte count <50 × 10^9^/L, precursor B cell phenotype, absence of a mediastinal mass, spinal fluid without blastic cells, and good response to the prednisone window. HR was defined as all patients not in the SR group.

### 2.3. Therapy

**Figure 1:**
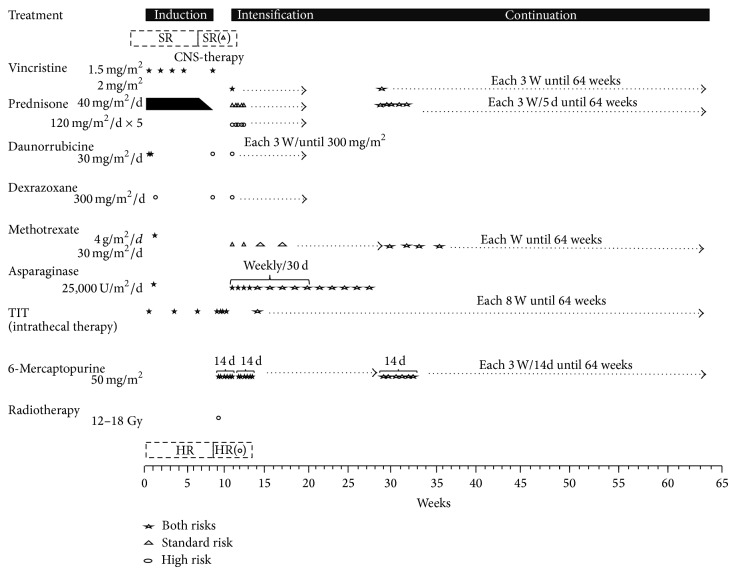
DCFI 00-01 therapy in paediatric patients with ALL who were younger than 16 years of age and who were treated at the paediatric haematology service of the specialist “La Raza” IMSS Medical Centre.

The treatment scheme is shown in Figure 1. Our patients have received the treatment mentioned previously since 1998. At that time, the classification was based on age and leucocyte count. In our hospital, the immunophenotype, cytogenetic study results, and molecular biology data were not available. In this report, we include those patients who were treated under the DFCI 00-01 protocol adapted to the local conditions without a window of investigation. All patients had been treated with a prednisone window comprising 60 mg/m^2^ SC for 7 days and intrathecal chemotherapy with methotrexate on day 0, and the induction to remission was 4 weeks. The anthracycline drug was daunorubicin instead of doxorubicin. CNS therapy was applied once complete haematological remission was reached. Afterwards, all patients received triple CNS intrathecal chemotherapy two times per week in four dosages adjusted according to the patient's age. The HR patients with major risk of relapse also received radiotherapy (12 Gy) to the brain.

All patients continued the intensification phase for 30 weeks. For the SR group, vincristine 2 mg/m^2^ at day 1 and each 3 weeks, asparaginase from* Escherichia coli* 25,000 UI/m^2^, and methotrexate 40 mg/m^2^ by intramuscular injection each week and 1 day after asparaginase were administrated. Prednisone was administered orally at 40 mg/m^2^ for 5 days and 6-mercaptopurine 50 mg/m^2^/day for 14 days. The HR group received additional intravenous daunorubicin 30 mg/m^2^ at day 1 and each three weeks. The stopping dose of daunorubicin was 300 mg/m^2^, dexrazoxane 300 mg/m^2^ before daunorubicin, and prednisone 120 mg/m^2^/day for 5 days. In patients who exhibited an allergic reaction to* E. coli* asparaginase, the treatment was suspended with no other option because another form of asparaginase was not available in our service. The maintenance was for 64 weeks. Either the SR or HR group the patients had received vincristine, prednisone, 6-mercaptopurine, and methotrexate equally than the intensification, but the HR group had received prednisone to 40 mg/m^2^ and intrathecal chemotherapy with triple drug every 8 weeks until the treatment was completed.

### 2.4. Response and Relapse Criteria

The prednisone response was determined using the absolute count of blasts in the peripheral blood on day 8 after 7 days of prednisone and one dose of intrathecal methotrexate on day 1. A poor response to prednisone (PRP) was defined as a blast count of ≥1 × 10^9^/L, and a good response to prednisone (GRP) was defined as a blast count of <1 × 10^9^ blasts. The response of the bone marrow to the induction therapy was evaluated on days 14 and 28. Complete remission was defined as <5% of blasts in bone marrow and without extramedullary disease.

Relapse was defined as an emergence of the disease with ≥25% of blasts in either bone marrow or extramedullary or both. Early death was defined as the death of a patient before evaluation of the remission stage on day 28 or 35 after the induction of remission. Event-free survival was defined as the time to the occurrence of resistance, relapse, death, or a second neoplasm. Global survival was defined as the time in months from the diagnosis to death from any cause or the last contact with the patient in the outpatient clinic or hospital.

### 2.5. Statistical Analysis

Qualitative variables are presented as absolute numbers or percentages. Quantitative variables are presented as a median value, as a measure of central tendency, and as a range between the minimum and maximum values. For quantitative variables without a normal distribution, the medians were compared using the Mann-Whitney *U* test for two independent groups. For qualitative variables, the chi-square or Fisher's exact test were used. *P* < 0.05 was considered to indicate that the difference had a low probability of a random error. The prednisone response was evaluated using a Kaplan-Meier survival analysis and the groups were compared using the log-rank test. Confounding variables in the analysis of the prednisone response were analysed using the Cox proportional-hazard model. All analyses were performed using SPSS statistical package version 21. Two analyses of survival were performed after 3 years and 5 years of follow-up. All of the children had complete data for the 3-year follow-up, but only 158 (52.3%) had data for the 5-year follow-up.

## 3. Results

A total of 302 children met the inclusion criteria and were included during the study period. There were more boys (53.3% boys). The median age was 7 years, and most of these patients were 1–5-year-old (40.4%). Most patients (92.4%) were classified with the precursor B phenotype.

The most frequent leucocyte count in these patients was <10 × 10^9^/L; the median was 10,790 × 10^9^/L. Interestingly, at the time of diagnosis, only 2.6% had CNS infiltration even though most patients (51.7%) were classified as HR.

Most of the patients had an initial good response to prednisone (80.1%) (Table 1), and 90.4% of these patients had a complete remission. The median follow-up was 3.9 years. The frequency of early mortality was 7%, and the frequency of relapse was 26.2%. Most cases of relapse were early relapse, mainly in the bone marrow (18.2%). The global mortality was 36.1% (Table 2). Because all patients had completed the 3 years of follow-up, the presence of events for relapse and death was estimated for 3 and 5 years.

### 3.1. Three-Year Follow-Up (Tables 3, 4, and 5)

Relapse was more frequent in the boys (24.6%). However, the girls had a higher frequency of events at 42.2%.

The highest relapse frequency was observed in the group aged 5.1–9.9 years (25.8%), who experienced a high frequency of events (47%). The age group with the highest mortality was the group aged >10 years (36.3%; RR 1.70, 95% CI 1.0–2.8).

The patients with the T cell immunophenotype had the highest frequency of death (47.8%; RR 2.1, 95% CI 1.1–4.1).

The SR patients had higher frequencies of relapse (25.3%) and events (42.5%). Most relapses (28.3%) occurred in patients with a leucocyte count >100 × 10^9^/L. This group of patients also had the highest frequency of events (50%; RR 1.84, 95% CI 1.0–3.3). However, 37.5% of the patients who died had a range of leucocyte count of 10–20 × 10^9^/L.

### 3.2. Five-Year Follow-Up

Boys had a higher frequency of relapse (30.5%), but there was no difference between boys and girls for the frequency of events. The frequency of death was 38.5% in the girls (RR 1.41; 95% CI 1.0–2.1).

The highest frequency of relapse was in the group age >10 years (75.2%) and this group had the highest frequency of events (58.7%).

The highest frequency of relapse was observed in patients with leukaemia of B cell lineage (26.9%). The mortality rate was highest in patients with T cell leukaemia (52.2%; RR 1.93, 95% CI 1.0–3.7).

The SR group had a higher frequency of relapse (30.1%) and higher frequency of events (47.9%). The highest frequency of relapse was observed in the group with a leucocyte count >100 × 10^9^/L (30.4%). This group also had the highest frequency of events (58.7%; RR 2.02, 95% CI 1.2–3.5) and highest mortality (45.7%; RR 1.86, 95% CI 1.0–3.7).

The factor that was most strongly associated with the occurrence of events at the 5-year follow-up was a leucocyte count >100 × 10^9^/L. Leucocyte count and the T cell immunophenotype were also associated with a higher risk of mortality at 5 years.

Of the 302 patients, 21 (7%) died during induction to remission (IR) and 43 (14.2%) died during complete remission. The causes of death were sepsis (65, 21.5%), haemorrhage (9, 8.7%), pneumonia (7, 6.7%), and typhlitis (4, 3.8%). The global mortality was of 36.1%, and 13 (12.5%) died in leukaemic activity. Toxicity associated with asparaginase was observed in 29% of all patients. The most common toxicity was an allergic reaction in 19%, pancreatitis in 7%, thrombosis in 3%, and only one involving the CNS. All of these reactions led to a definitive suspension of the drug.

## 4. Discussion

In our patient population, which was treated using the DFCI 00-01 protocol, after a median follow-up of 3.9 years, the global survival and event-free survival rates were lower than those obtained from the DFCI Consortium reports in a 5-year follow-up (63.9% and 52.3% versus 90% and 81%, resp., for the group treated with prednisone). There were other differences between our patients and those reported by other groups. For example, in our study, most patients were in the HR group [7–11], which may have influenced our results. The high mortality rate during induction (7%) may be associated with the delayed arrival of the patient at our hospital, bad conditions, tumour load, malnutrition, presence of infection, or toxicity of the treatment [24]. Our results are similar to those reported previously by another centre in Mexico [25] and by a multicentre study in Central America (AHOPCA and Pacheco et al.) [26, 27], which reported mortality rates of 3% and 7% during IR. In our study, 80.1% showed a prednisone good response (PGR), whereas >90% of patients showed a PGR in two other studies (8, 29). To our knowledge, no studies have reported on a diminished response to steroid in the Hispanic population.

Other factors were hyperleukocytosis (15.2%), which has been reported previously. When the leucocyte count is >200 × 10^9^/L [28, 29], the toxicity of the treatment is associated with a high percentage of deaths during complete remission (14.2%) and with the presence of an infectious disease leading to septic shock (21.5%).

A higher frequency of relapse (26.2%) was observed in our patients compared with other studies [22, 23, 29]. The factors associated with relapse at the 3- and 5-year follow-ups in our population were gender (male), age (5.1–9.9 years), B cell lineage, leucocyte count >100 × 10^9^/L), and SR. By contrast, there was a protective effect in the HR group (RR 0.83, 95% CI 0.5–1.4). This finding suggests that determining the risk group using the NCI criteria may be imprecise and that the correct classification of risk is needed for the proper application of therapy to increase the chance that the patient can remain free of events. If precise data are not available (i.e., cytogenetic or molecular biology), a third classification of intermediate risk, as noted previously [5], may be needed. This third classification is based on minimal residual disease (MRD), determined by polymerase chain reaction (PCR) at two times, which redefines all prognostic factors. Attarbaschi et al. [30] showed that only the MRD was reliable enough to discriminate the SR and HR of relapse in a group of patients with ALL of the precursor B cell phenotype (*P* = 0.02).

In our population, most of the relapses occurred early and were most frequent in bone marrow (18.2%). Studies of MRD have shown that the leukaemic cells found during relapse originate from residual cells and are resistant to chemotherapy [31–33], as was observed in our results (i.e., a high percentage of deaths (12.5%) were associated with leukaemic activity). Other factors that could have influenced the relapse rate in our study were the use of methotrexate (4 g/m^2^) in a 1 h infusion followed by generous doses of leucovorin, because the levels of monitoring were not available. Mikkelsen et al. [34] reported that shortening the time of infusion of methotrexate reduced the accumulation of active methotrexate in the leukaemic cells and therefore the antileukaemic effect is reduced. Skärby et al. [35] reported that high doses of leucovorin during the treatment with high doses of methotrexate reduced the average cure rate in children with ALL.

The rate of isolated CNS relapse was low (2.6%) and was associated with the intensive use of directed therapy to the CNS (3 IT in IR, 4 in the 2 weeks of the therapy directed to the CNS in a total of 12 until the 24 months of treatment were completed, joined to radiotherapy in the patients with HR) and the systemic chemotherapy, as was proven in clinical assays in the groups of leukaemia: CCG [7], BFM [8], and St. Jude [9]. These studies have shown that if additional therapy is directed to the CNS early, it is possible to replace radiotherapy, which may be reserved only for those patients with a higher risk of relapse or with CNS3 status, because of its secondary effects, which have been observed in long-term survivors of ALL after either chemotherapy or radiotherapy [36–38].

The factors more strongly associated were ages <1 year and >10 years, leucocyte count >100 × 10^9^/L, and T cell phenotype. These factors have also been identified previously as indicators of poor prognosis [23, 28, 29, 39–41]. In our study, the factor most strongly associated with the occurrence of events was a leucocyte count >100 × 10^9^/L. We used this criterion to classify our patients as very high risk and then treated them with more intensive chemotherapy. Our poor results were associated with toxic death during induction, complete remission during treatment, and relapse. Toxicity remains as a main obstacle to the success of treatment of our population. Our results are similar to those reported from other Central American countries (Nicaragua, Costa Rica, El Salvador, Honduras, and Panama) by the AHOPCA ALL 2008 study [26], in which the treatment was based on local regimens adapted from the BFM group to the local situation. We now have two projects underway to reduce the frequency of toxicity in children with ALL, especially in malnourished children.

Several groups have observed that the results of the treatment of children with ALL depend on the biological diversity of the leukaemic cells, the multidrug treatment scheme, and individual variability in the metabolism of the drugs. However, in developing countries, socioeconomic status is also a strong predictor that is independent of the relapse and mortality associated with the treatment [42, 43]. In Mexico, this can be an important factor because most of our patients live far from the treatment centres, which causes delays in the timely management of their disease. Bhatia et al. [44] showed that the lack of adherence to mercaptopurine increased the risk of relapse in Hispanic patients compared with non-Hispanics. Recently, polymorphisms such as ARID5B have been associated with a higher incidence of ALL and greater risk of relapse in Hispanic patients, a finding that may explain the higher incidence and worse results in Hispanic patients [45, 46]. Additionally, there arrangement of CRLF2 associated with mutations in JAK and alterations in IKZF1 in Hispanics may be responsible for the poor response of ALL of B cell lineage [47].

To improve our results, we decided that, during the induction phase, methotrexate should be given at a dose of 1 g/m^2^ infused for 24 h instead of 4 g/m^2^ infused for 1 h, and we then changed the dose of leucovorin to 25 mg/m^2^ SC to 48 and 52 h. And the next dosage should be given according to the level of methotrexate. To improve the stratification of risk, we suggest redefining the risk groups by introducing an intermediate risk group, another HR group, and a very HR group. We also recommend promoting the implementation of reference centres for the cytogenetic and molecular diagnosis of MRD and the formation of national cooperative groups linked to international groups.

We consider that protocol DFCI 00-01 is an excellent of chemotherapy scheme. With the exception of induction, all of the treatments can be applied to outpatients, which will decrease the cost. However, the disadvantage for low- or middle-income countries such as Mexico is the lack of alternatives for asparaginase, which could contribute to the relapse observed in some patients when asparaginase is suspended.

## Figures and Tables

**Table 1:** Demographic and baseline characteristics of the study population (*N* = 302).

Characteristic	*n*	%	Median (m/m)
Total	**302**	**100**	
Male	167	53.3	
Female	135	44.7	
Age (years)			7 (<1–15)
Age groups (years)			
<1	1	0.3	
1–5	122	40.4	
5.1–9.99	66	21.9	
≥10	113	37.4	
Immunophenotype			
B	279	92.4	
T	23	7.6	
Leucocyte count (1 × 10^9^/L)			10,790 (720–939,830)
Groups of leucocyte count			
<10	147	48.7	
10–20	48	15.9	
20–50	34	11.3	
50–100	27	8.9	
>100	46	15.2	
NCS infiltration	8	2.6	
Testicular infiltration	4	1.3	
Prednisone response			
Good	242	80.1	
Poor	60	19.9	
SR	146	48.3	
HR	156	51.7	

(m/m): minimum/maximum.

**Table 2:** Treatment responses (*N* = 302).

	*n*	%	median (m/m)
Complete remission	273	90.4	
Failure	8	2.6	
Early death	21	7.0	
Treatment dropout	6	2.0	
Relapse			
No	223	73.8	
Yes	79	26.2	
Time of relapse			
Very early	27	8.9	
Early	40	13.2	
Late	12	4.0	
Site of relapse			
Bone marrow (BM)	55	18.2	
NCS	8	2.6	
BM + NCS	13	4.3	
Testicle	3	1.0	
Dead	193	63.9	
Alive	109	36.1	
Duration of follow-up (years)			3.9 (7 days–7.3)

m/m: minimum/maximum.

**Table 3:** Relapses and deaths in paediatric patients with ALL who were younger than 16 years of age and who were treated at the paediatric haematology service of the specialist “La Raza” IMSS Medical Centre.

*N* = 302	Total	Relapse (at 3 years)	Not Relapse (at 3 years)	Relapse (at 5 year)	No Relapse (at 5 years)	Death (at 3 years)	Lives (at 3 years)	Death (at 5 years)	Lives (at 5 years)
	*n* (%)	*n* (%)	*n* (%)	*n* (%)	*n* (%)	*n* (%)	*n* (%)	*n* (%)	*n* (%)
Sex									
Male (%)	167 (100)	41 (24.6)	126 (75.4)	51 (30.5)	116 (69.5)	48 (28.7)	119 (71.3)	54 (32.3)	113 (67.7)
Female (%)	135 (100)	26 (19.3)	109 (80.7)	28 (20.7)	107 (79.3)	44 (32.6)	91 (67.4)	52 (38.5)	83 (61.5)
Age (years)									
<1	1 (100)	1 (100)	0 (0.0)	1 (100)	0 (0.0)	0 (0)	1 (100)	1 (100)	0 (0.0)
1–5	122 (100)	25 (20.5)	97 (79.5)	31 (25.4)	91 (74.0)	29 (23.8)	93 (76.2)	35 (28.7)	87 (71.3)
5.1–9.99	66 (100)	17 (25.8)	49 (74.2)	19 (28.8)	47 (71.2)	22 (33.3)	44 (66.7)	26 (39.4)	40 (60.6)
≥10	113 (100)	24 (21.2)	89 (78.7)	28 (24.8)	85 (75.2)	41 (36.3)	72 (63.7)	44 (38.9)	69 (61.1)
Lineage									
B	279 (100)	73 (22.6)	216 (77.4)	75 (26.9)	204 (73.1)	81 (29.0)	198 (71.0)	94 (33.7)	185 (66.3)
T	23 (100)	4 (17.4)	19 (82.6)	4 (17.9)	19 (82.6)	11 (47.8)	12 (52.2)	12 (52.2)	11 (47.8)
Risk group									
Standard	146 (100)	37 (25.3)	109 (74.7)	44 (30.1)	102 (69.9)	44 (30.1)	102 (69.9)	48 (32.9)	98 (67.1)
High	156 (100)	30 (19.2)	126 (80.8)	35 (22.4)	121 (77.6)	48 (30.8)	108 (69.2)	58 (37.2)	98 (62.8)
WBC (1 × 10^9^/L)									
<10	147 (100)	34 (23.1)	113 (76.9)	38 (25.9)	109 (74.1)	40 (27.2)	107 (72.8)	43 (29.3)	104 (70.7)
10–20	48 (100)	10 (20.8)	38 (79.2)	14 (29.2)	34 (70.8)	18 (37.5)	30 (62.5)	20 (41.7)	28 (58.3)
20–50	34 (100)	3 (8.8)	31 (91.2)	5 (14.7)	29 (85.3)	11 (32.4)	23 (67.6)	12 (35.3)	22 (64.7)
50–100 000	27 (100)	7 (25.9)	20 (74.1)	8 (29.6)	19 (70.4)	6 (22.2)	21 (77.8)	10 (37.0)	17 (63.0)
≥100	46 (100)	13 (28.3)	33 (71.7)	14 (30.4)	32 (69.6)	17 (37.0)	29 (63.0)	21 (45.7)	26 (54.3)

**Table 4:** Events and deaths in paediatric patients with ALL who were younger than 16 years of age and who were treated at the paediatric haematology service of the specialist “La Raza” IMSS Medical Centre.

Characteristic	Event				Death			
	3 years		5 years		3 years		5 years	
	No	Yes	No	Yes	No	Yes	No	Yes
	*n* (%)	*n* (%)	*n* (%)	*n* (%)	*n* (%)	*n* (%)	*n* (%)	*n* (%)
Sex								
Male	99 (59.3)	68 (40.7)	89 (53.3)	78 (46.7)	119 (71.3)	48 (28.7)	113 (67.7)	54 (32.3)
Female	78 (57.8)	57 (42.2)	72 (53.3)	63 (46.7)	91 (67.4)	44 (32.6)	83 (61.5)	52 (38.5)
Age (years)								
<1	0 (0)	1 (100)	0 (0)	1 (100)	1 (100)	0 (100)	0 (0)	1 (100)
1–5	79 (64.8)	43 (35.2)	71 (58.2)	51 (41.8)	93 (76.2)	29 (23.8)	87 (71.3)	35 (28.7)
5.1–9.99	35 (53.0)	31 (47.0)	32 (48.5)	34 (51.5)	44 (66.7)	22 (33.3)	40 (60.6)	26 (39.4)
≥10	63 (55.8)	50 (44.2)	58 (51.3)	55 (48.7)	72 (63.7)	41 (36.3)	69 (61.1)	44 (38.9)
Immunophenotype								
B	166 (59.5)	113 (40.5)	150 (53.8)	129 (46.2)	198 (71.0)	81 (29.0)	185 (66.3)	94 (33.7)
T	11 (47.8)	12 (52.2)	11 (47.8)	12 (52.2)	12 (52.2)	11 (47.8)	11 (47.8)	12 (52.2)
WBC count (1 × 10^9^/L)								
<10	91 (61.9)	56 (38.1)	87 (59.2)	60 (40.8)	107 (72.8)	40 (27.2)	104 (70.7)	43 (29.3)
10–20	26 (54.2)	22 (45.8)	21 (43.8)	27 (56.3)	30 (62.5)	18 (37.5)	28 (58.3)	20 (41.7)
20–50	20 (58.8)	14 (41.2)	18 (52.9)	16 (47.1)	23 (67.6)	11 (32.4)	22 (64.7)	12 (35.3)
50–100	17 (63.0)	10 (37.0)	16 (59.3)	11 (40.7)	21 (77.8)	6 (22.2)	17 (63.0)	10 (37.0)
>100	23 (50.0)	23 (50.0)	19 (41.3)	27 (58.7)	29 (63.0)	17 (37.0)	25 (54.3)	21 (45.7)
Risk group								
Standard	84 (57.5)	62 (42.5)	76 (52.1)	70 (47.9)	102 (69.9)	44 (30.1)	98 (67.1)	48 (32.9)
High	93 (59.6)	63 (40.4)	85 (54.3)	71 (45.5)	108 (69.2)	48 (30.8)	98 (62.8)	58 (37.2)

**Table 5:** Cox proportional-hazards model. Disease-free survival and death in paediatric patients with ALL who were younger than 16 years of age and who were treated at the paediatric haematology service of the specialist “La Raza” IMSS Medical Centre.

Characteristic	DFS 3 years			DFS 5 years			Death 3 years			Death 5 years		
	RR	95% CI	*P*	RR	95% CI	*P*	RR	95% CI	*P*	RR	95% CI	*P*
Sex												
Male												
Female	1.202	0.839–1.721	0.316	1.137	0.811–1.595	0.457	1.307	0.861–1.984	0.208	1.413	0.956–2.088	0.082
Age (years)												
<1	4.288	0.531–34.611	0.172	3.947	0.495–31.454	0.195	0.000	0.000	0.000	3.616	0.446–29.352	0.229
1–5	1			1			1			1		
5.1–9.99	1.504	0.942–2.403	0.087	1.384	0.891–2.148	0.148	1.556	0.887–2.728	0.123	4.573	0.940–2.633	0.085
>10	1.464	0.958–2.239	0.078	1.358	0.912–2.021	0.132	1.699	1.036–2.787	0.036	1.561	0.982–2.479	0.060
Leucocytes (1 × 10^9^/L)												
<10												
10–20	1.302	0.792–2.140	0.297	1.489	0.942–2.354	0.089	1.437	0.821–2.516	0.205	1.495	0.876–2.550	0.140
20–50	1.193	0.655–2.175	0.564	1.253	0.712–2.205	0.435	1.286	0.650–2.545	0.470	1.322	0.688–2.541	0.402
50–100	1.133	0.520–2.470	0.753	1.167	0.556–2.449	0.683	0.990	0.390–2.510	0.982	1.374	0.620–3.044	0.434
>100	1.842	1.033–3.285	0.038	2.025	1.172–3.496	0.011	1.604	0.833–3.088	0.158	1.860	1.011–3.422	0.046
Phenotype												
B												
T	1.676	0.893–3.145	0.108	1.479	0.794–2.754	0.218	2.071	1.054–4.070	0.035	1.930	1.017–3.664	0.044
Risk												
Standard												
High	0.765	0.481–1.216	0.258	0.753	0.485–1.169	0.206	0.823	0.487–1.388	0.464	0.830	0.503–1.371	0.467

DFS: disease-free survival; RR: relative risk; CI: confidence interval.
